# Immunohistochemical properties of embryonic telocytes in a myogenic microenvironment

**DOI:** 10.1038/s41598-024-62103-1

**Published:** 2024-05-27

**Authors:** Soha A. Soliman

**Affiliations:** Department of Histology, Faculty of Veterinary Medicine, South Valley University, Qena, Egypt

**Keywords:** Cell biology, Electron microscopy

## Abstract

Telocytes are a unique interstitial cell type that functions in adulthood and embryogenesis. They have characteristic immunohistochemical phenotypes while acquiring different immunohistochemical properties related to the organ microenvironment. The present study aims to investigate the immunohistochemical features of embryonic telocytes during myogenesis and describe their morphology using light microscopy and TEM. Telocytes represent a major cellular constituent in the interstitial elements. They had distinguished telopodes and podoms and formed a 3D interstitial network in the developing muscles. They formed heterocellular contact with myoblasts and nascent myotubes. Telocytes also had distinctive secretory activity. Telocytes identified by CD34. They also express CD68 and MMP-9 to facilitate the development of new tissues. Expression of CD21 by telocytes may reveal their function in immune defense. They also express VEGF, which regulates angiogenesis. In conclusion, the distribution and immunological properties of telocytes in the myogenic tissue indicate that telocytes provide biological and structural support in the development of the myogenic tissue architecture and organization.

## Introduction

Interstitial connecting cells known as telocytes. Numerous cell types and structures can make cellular contact with telocytes due to their unique structural character^1^. The cell prolongations or telopodes that telocytes develop can grow to be hundreds of microns long. They make up a large 3D interstitial network. Telopodes consist of dilated segments called podoms and narrow segments called podomers, which contain aggregates of mitochondria and endoplasmic reticulum^2^.

According to the gene expression data, telocytes have a role in tissue homeostasis, remodeling^3^, angiogenesis^3^, signaling within cells^3,4^, cell growth and mobility^4^, suppression of oxidative stress and cellular aging^5^, and anti-inflammatory and anti-oncogenic role^6^.

Cell–cell communication is regarded as a key telocyte characteristic. For telocytes, two cell communication mechanisms have been identified: the paracrine pathway and cell contact. Several types of cell contact are documented between telocytes and other cells, including minute junctions like point, nano, and planar contacts as well as cell contact^7^. Three different forms of cell contact could be formed by telocytes: gap junction, adherence (puncta adherentes minima and processes adherentes), and direct opposition. A gap junction enables the movement of signals between cells^7,8^. Telocytes use secretory vesicles, exosomes, ectosomes, and multivesicular vesicles to transport active chemicals to effector cells via a paracrine pathway^2,9, 10^.

One type of tissue generated from mesoderm is skeletal muscle. Mesenchymal cells are stimulated to develop into myogenic cell lines and produce myoblasts, which are the precursors of muscle. This process starts with myogenic differentiation. For skeletal myogenesis, a fusion of myoblasts to generate multinucleated syncytia is a typical event. When a myotube grows, individual skeletal myoblasts often combine to form the final multinucleated skeletal structure^11^. Recent investigations explore the role of telocytes in skeletal muscle development^12^. However, there is a lack of immunological characteristics in telocytes. The current study investigated the immunological properties of telocytes associated with skeletal myogenic development.

## Results

### Morphology of telocytes in the developing muscles using histochemical stains

**Figure 1:**
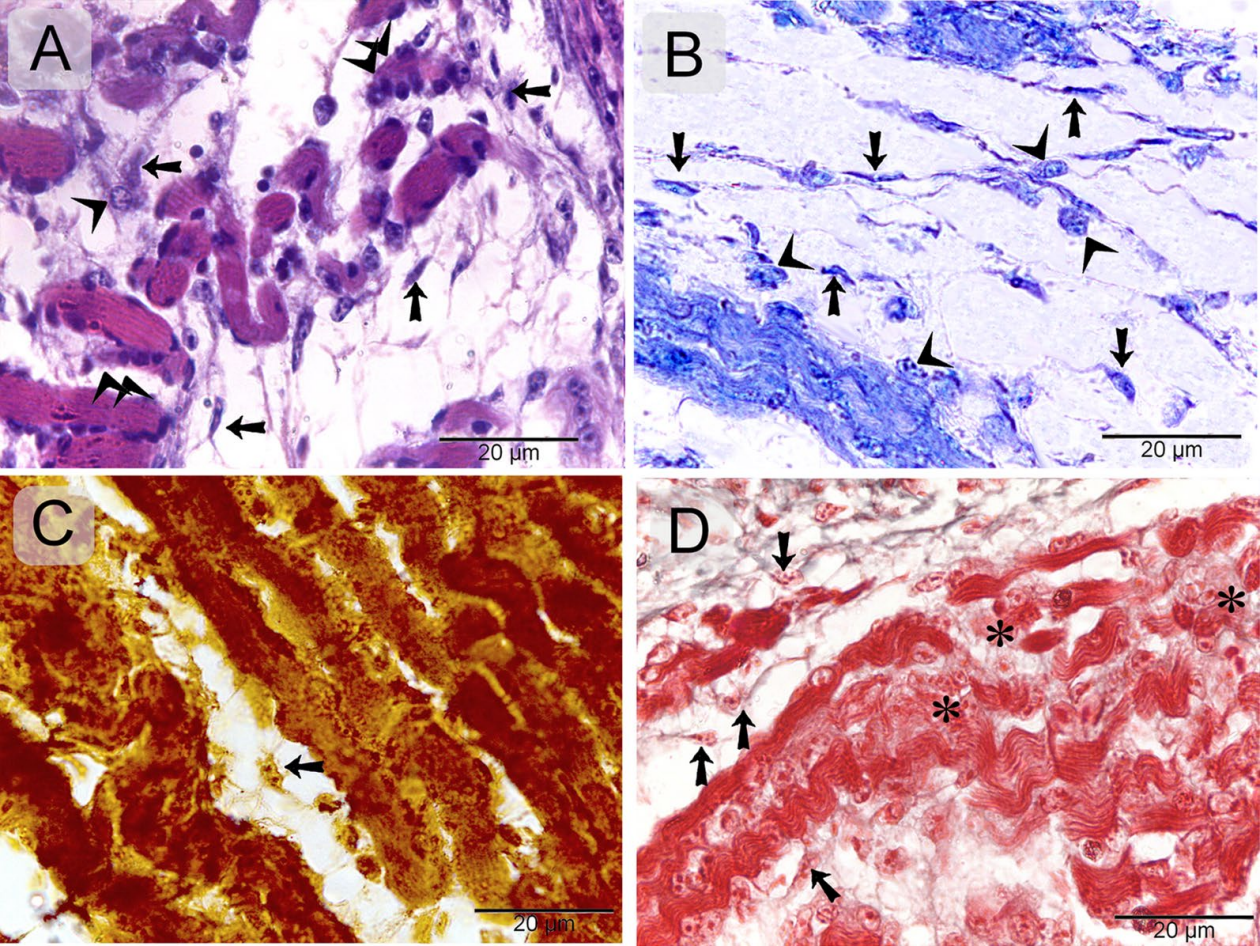
Recognition of telocytes in of the emryonic skeletal muscles using histochemical stains. Paraffin sections of the Caudofemoralis muscles of quail embryos stained with H&E (**A**), methylene blue (**B**), Grimelius’s silver nitrate method (**C**), Crossman’s trichrome (**D**). Telocytes (arrows) formed 3D network within the developing muscles. Note nascent myotube (double arrowheads), myoblast (arrowheads), and cytoplasmic areas of the developing myogenic cells contained scant myofibrils (asterisks).

Recognition of telocytes in the embryonic skeletal muscles using histochemical stains, including H&E (Fig. 1A), methylene blue (Fig. 1B), Grimelius’s silver nitrate method (Fig. 1C), and Crossman’s trichrome (Fig. 1D), Telocytes formed a 3D network around the nascent myotube and myoblasts.

### Identification of telocytes in the developing skeletal muscles using semithin sections

**Figure 2:**
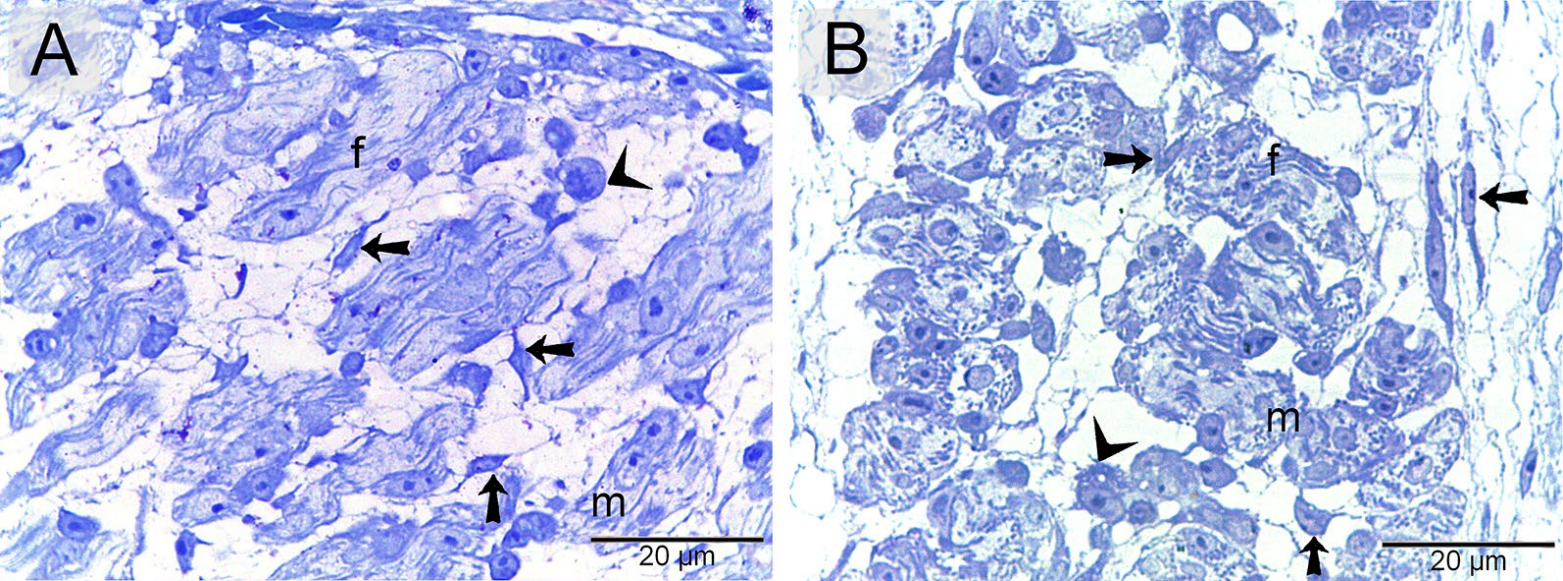
Recognition of telocytes in of the emryonic skeletal muscles using semithin sections. Semithin sections stained with toluidine blue (**A**) and methylene blue (**B**). Telocytes (arrows) formed 3D network within the developing muscles. Note nascent myotube (m), myoblast (arrowheads), developing myofibrils (f).

Telocytes were identified using toluidine blue (Fig. 2A) and methylene blue (Fig. 2B). They formed a 3D network within the developing muscles. Telocytes were observed around the nascent myotube and myoblast.

### Identification of telocytes in the developing muscles using TEM

**Figure 3:**
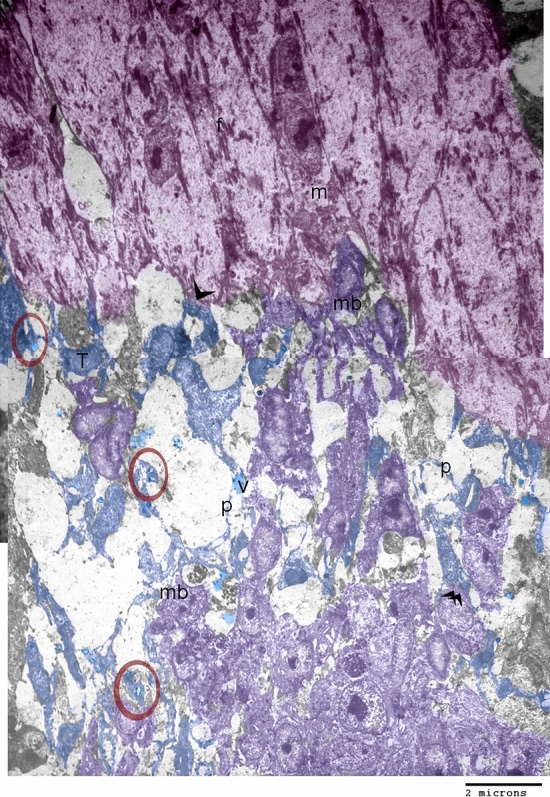
Identification of telocytes in the developing muscles using TEM. Colored ultrathin section. Telocytes (blue colored) composed of cell body (T) and podmeres. Telocytes established direct contact with myotubes (arrowhead) and myoblast (double arrowhead). Note podoms (red circles), secretory vesicles (V), nascent myotubes (pink colored) that were actively synthesize the myofilaments, myoblast (violet colored).

Telocytes are composed of cell bodies and polymers, which have distinctive podoms. They formed a 3D network surrounding the myoblasts and the nascent myotubes that actively synthesized the myofilaments. Telocytes established direct contact with the myoblasts as well as the myotubes. They release secretory vesicles (Fig. 3).

### Immunohistochemical properties of telocytes

**Figure 4:**
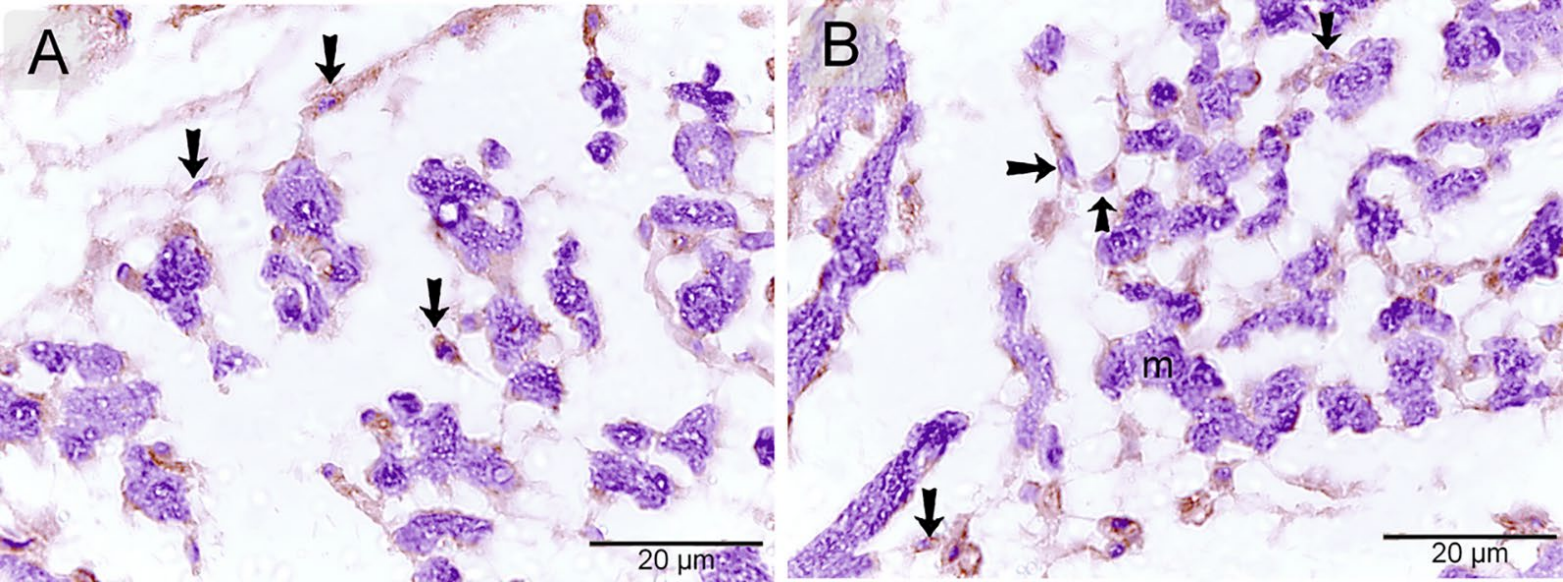
Identification of telocytes in the developing muscles using CD34. Paraffin sections immuneostained for CD34. CD34-positive telocytes (arrows) formed 3D network within the developing muscles. Note nascent myotube (m).

**Figure 5:**
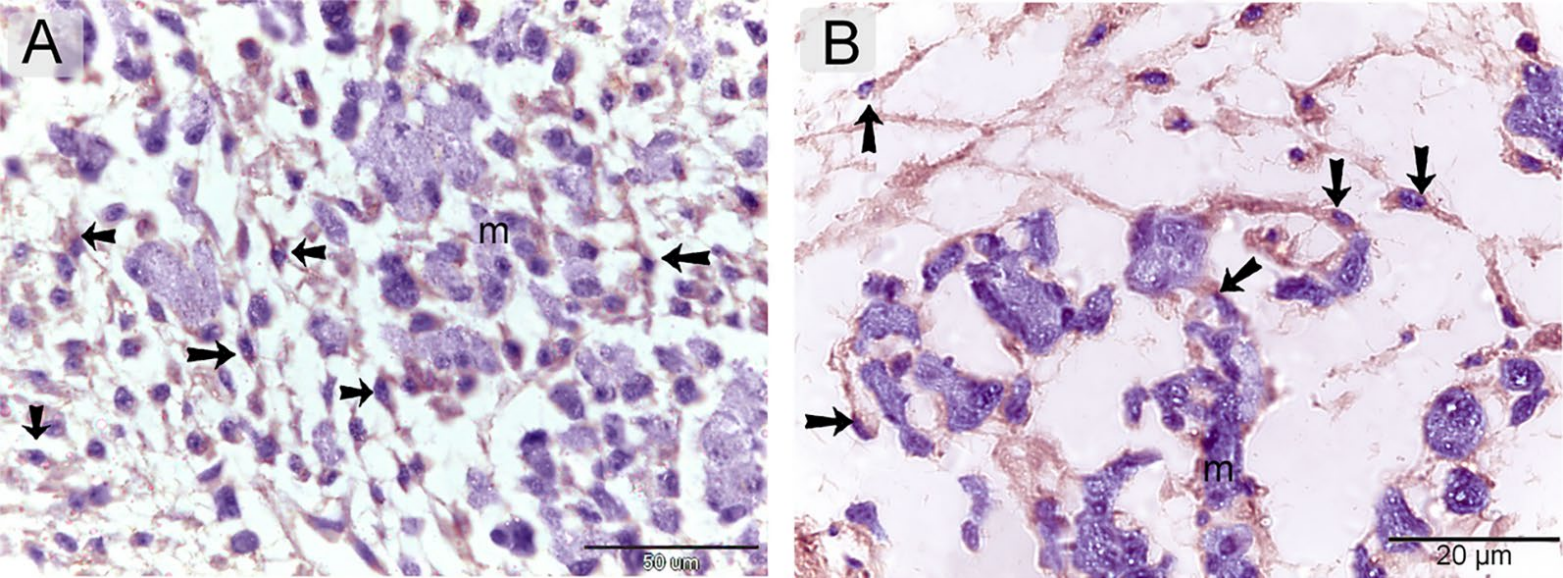
Immunohistochemical staining of the developing muscles using VEGF. Paraffin sections immuneostained for VEGF. VEGF-positive telocytes (arrows) formed 3D network within the developing muscles. Note nascent myotube (m).

**Figure 6:**
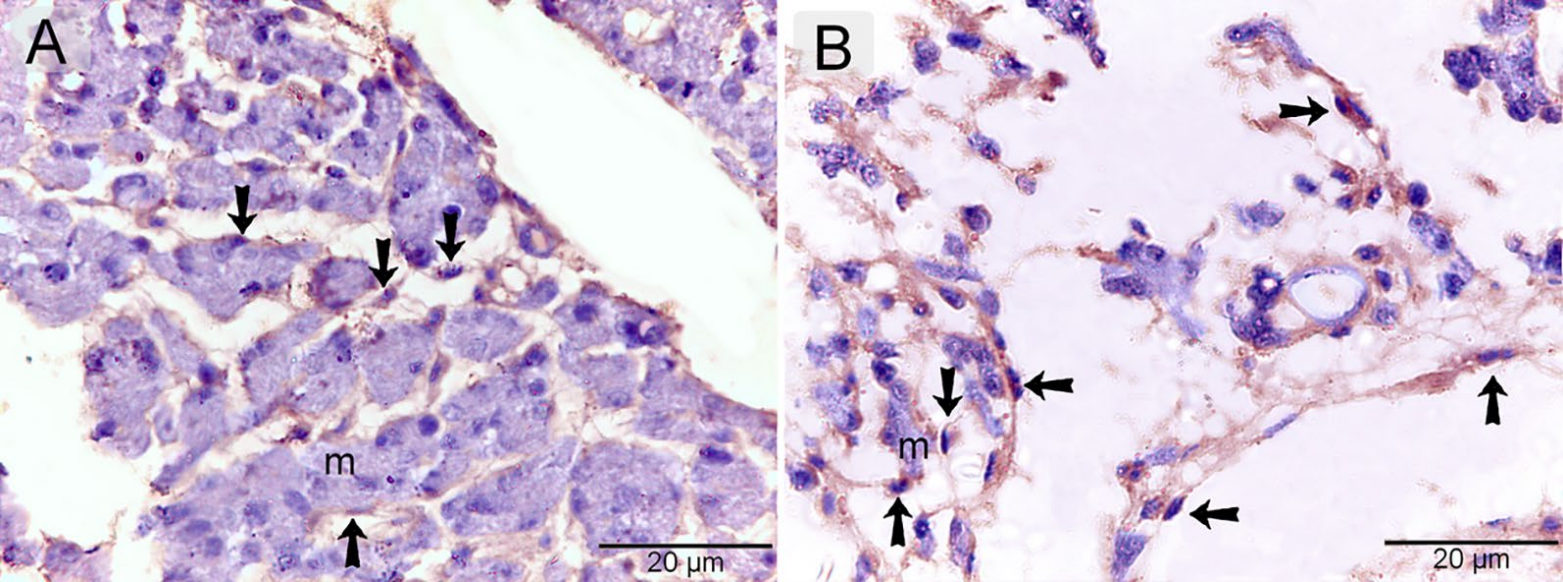
Immunohistochemical staining of the developing muscles using CD21. Paraffin sections immuneostained for CD21. CD21-positive telocytes (arrows) formed 3D network within the developing muscles. Note nascent myotube (m).

**Figure 7:**
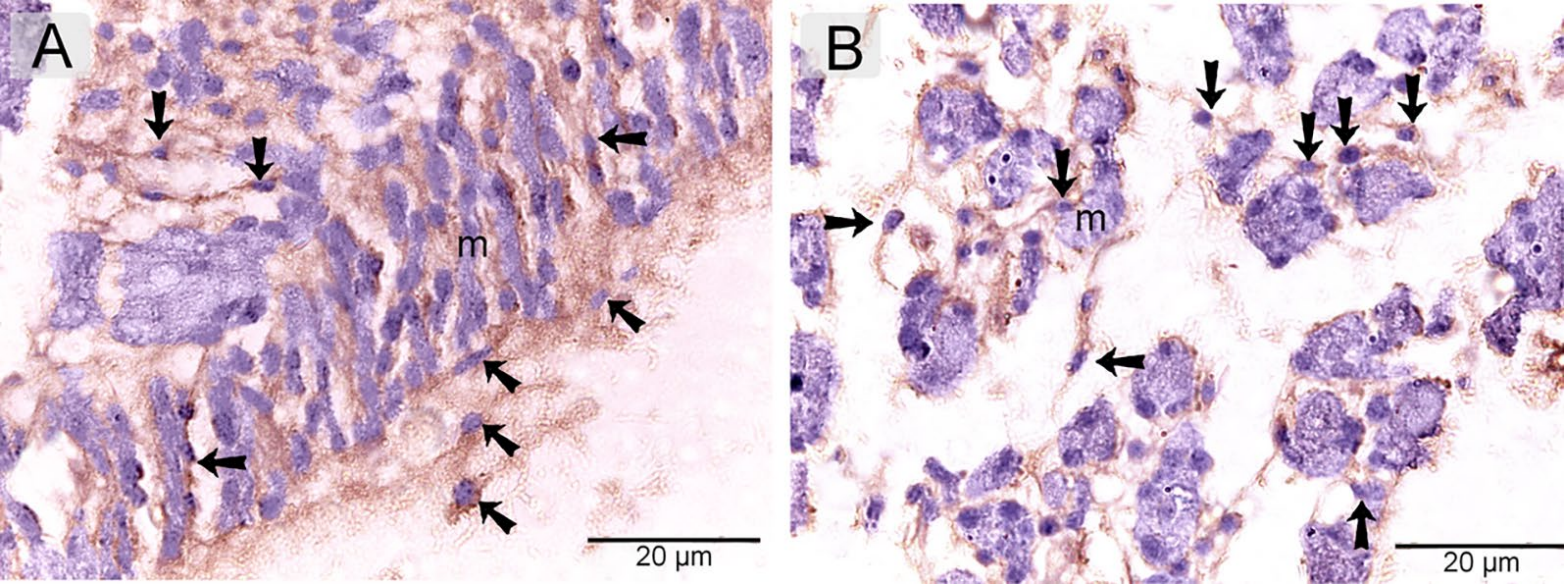
Immunohistochemical staining of the developing muscles using CD68. Paraffin sections immuneostained for CD68. CD68-positive telocytes (arrows) formed 3D network within the developing muscles. Note nascent myotube (m).

**Figure 8:**
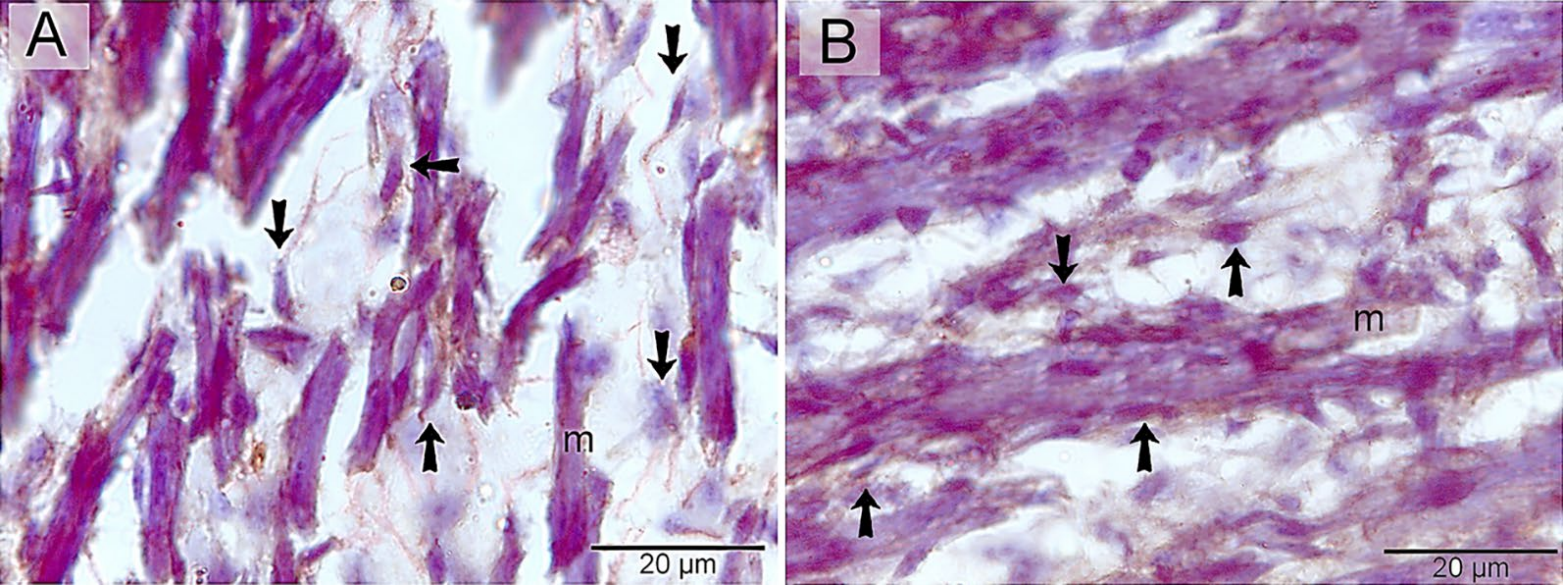
Immunohistochemical staining of the developing muscles using MMP-9. Paraffin sections immuneostained for MMP-9. MMP-9-positive telocytes (arrows) formed 3D network within the developing muscles. Note nascent myotube (m).

Telocytes were identified using CD34. CD34-positive telocytes formed a 3D network within the developing muscles (Fig. 4A, B). TCs exhibited strong immunoaffinity for VEGF (Fig. 5A, B), CD21 (Fig. 6A, B), CD68 (Fig. 7A, B), and MMP-9 (Fig. 8A, B).

**Figure 9:**
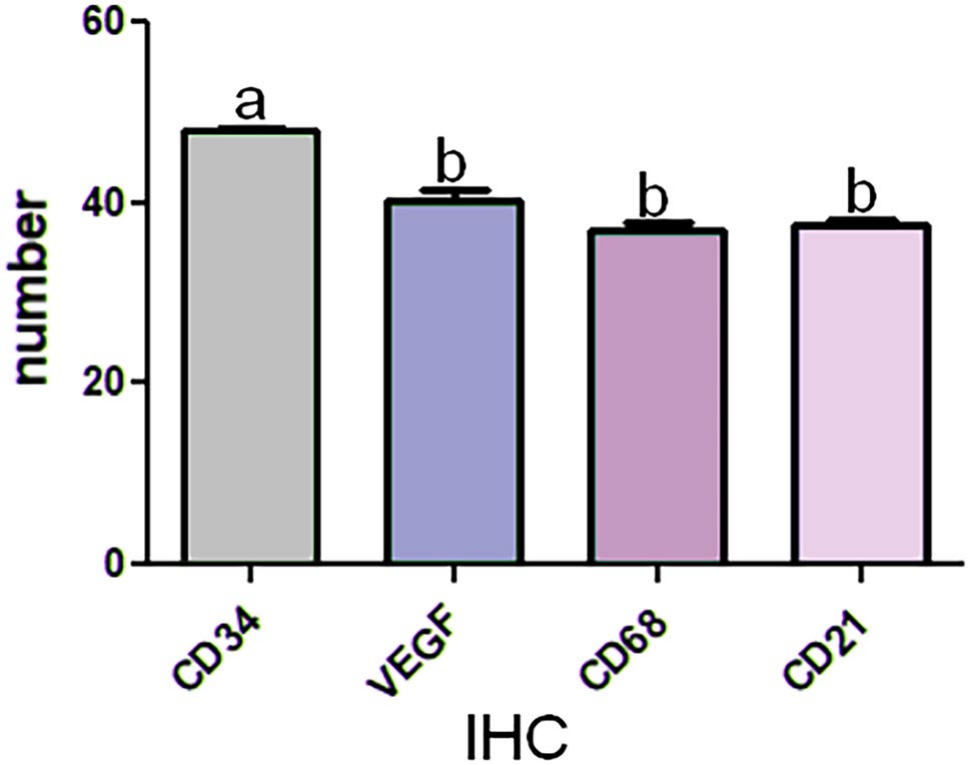
A column graphic representation of the mean numbers of positive CD34, VEGF, CD68, CD21 receptor TCs at 8 days embryo. Data are presented as the mean ± standard error (n = 8). IHC Expression with different lowercase letters are significantly different (one-way ANOVA: p < 0.05).

The number of CD34-positive TCs in the 8-day embryos group (mean ± SE) was 48.00 ± 1.309. The number of VEGF-positive TCs in the 8-day embryos group (mean ± SE) was 40.38 ± 0.5. The number of CD21-positive positive TCs in the 8-day embryos group (mean ± SD) was 37.38 ± 1.34. The number of CD68-positive positive TCs in the 8-day embryos group (mean ± SD) was 37.50 ± 1.512. The results showed that there was a significant difference between the CD34 receptor and other group receptors with (p < 0.05) (Fig. 9).

## Discussion

In the current study, telocytes are recognized by their unique podomeres and distinct podoms. Telocytes created a three-dimensional network and established direct contact with both myoblasts and nascent myotubes. These cells are actively synthesizing myofilaments. A previous study indicated that embryonic telocytes are widely distributed. They constitute an essential stromal component in the growing skeletal muscle and are arranged into an extensive structure that is dispersed throughout the epimysium, perimysium, and endomysium^13^.

In fetal muscle tissue, a reticular network is present which is closely associated with blood vessels, and primary and secondary myotubes. Telocytes networks are more numerous and show higher reactivity to CD34. In contrast, muscle tissue from 12 weeks of gestation shows a significant decrease in the quantity and immunopositivity of Telocytes, where mature myotubes are visible. Between nine and twelve weeks, there are similar changes in the quantity, density, and structure of the muscle stroma. Additionally, blood vessels become most abundant between 10 and 11.5 weeks. According to the authors, Telocytes may be essential throughout the early stages of myogenic development, possibly directing angiogenesis, tissue organization, compartmentalization, and myotube maturation^12^.

In the current investigation, it was found that TCs expressed MMP-9, which indicates proteolytic activity. MMP-9 helps remove extracellular matrix components that may hinder the migratory cells’ ability to enter the tissue and creates the new space required for the formation of new structures. MMPs are families of proteolytic enzymes that are essential for tissue remodeling and development as they stimulate the degradation of extracellular matrix components. Gelatinase B, or MMP-9, is a metal-dependent endopeptidase that promotes the growth and migration of cancer cells and is also involved in angiogenesis, as well as the activation of chemokines and cytokines^14^. MMP-9 can proteolytically degrade various ECM components such as collagen types IV, V, XIk', XIVl', elastin, aggrecan, link protein, decorin, laminins, entactin, SPARCq, myelin basic protein, 2Mn, 1Pli, IL-1, and proTNF^15^.

In the current study, a certain phagocytic marker was used to identify the properties of embryonic telocytes. The expression of CD68 is unique to phagocytic cells, which have formed endosomal-lysosomal systems. CD68, a member of the D-scavenger receptor family, is associated with the maturation of late endosomes and lysosomes. The granules of phagocytes containing CD68 are shown in the illustrations^16^.

The current study reveal that expression of CD68 of telocytes, which linked to phagocytic activities which may help in elimination of unnecessary components may be cellular or debris or degrade matrix components as a result of MMP-9 activity.

TCs express the marker specific for one of the immune cells, CD21. The CD21 endogenous ligand binds to complement component C3 fragments, interferon-alpha, and CD23. CD21 is an integral part of activated B- and T-lymphocytes. An integral part of activated B- and T-lymphocytes is CD21. Interferon, an antiviral cytokine, and DNA-DNA complexes (chromatin) are two innate immune receptors that CD21 interacts with^17^.

Expression of VEGF by TCs reveals the angiogenic role of TCs during development. Angiogenesis is the process by which blood vessels develop from the preexisting vasculature^18^. VEGF expression encourages the development of new blood vessels^19^. VEGF is a member of the platelet-derived growth factor family. VEGF plays several common roles in the development of angiogenesis^20^, vascular permeability^21^, and vascular integrity^22^. The current data revealed that Expression of VEGF development of the myogenic vasculature*.*

In conclusion, telocytes exhibit proteolytic activity in the extracellular matrix (ECM) by expressing MMP-9. On the other hand, expressing CD68 reveals the phagocytic activity of telocytes. Based on these findings, it can be inferred that telocytes exhibited both proteolytic and phagocytic activity, removing old components and creating new areas for the growth of myogenic tissues. Telocytes also express CD21, which has an essential role in immune function. Expression of VEGF by telocytes promotes the development of new vasculature. The current research indicates that TCs are a distinct cellular component of developing skeletal muscle and may play a role in myogenesis. These unique stromal cells may impact embryonic skeletal muscle tissue architecture and organization through their telopode network and participate in the development of myogenic tissue and angiogenesis.

## Material and methods

### Ethical approval

The National Ethics Committee of South Valley University and the veterinary authorities in Qena Province, Egypt, authorized the methodology utilized in this study. “All procedures were carried out in compliance with the applicable policies and guidelines.”

The Research Quail Farm, affiliated with the Department of Histology at the Faculty of Veterinary Medicine, South Valley University, Qena, Egypt, is the source of fertilized quail (Coturnix japonica) eggs that we have acquired. The fertilized eggs were incubated at 37.5 °C and 65% relative humidity. The eggs were automatically rotated every six hours following the third day of incubation. Fertilized eggs were removed on the eighth day of incubation and kept for 4 h at − 20 °C before the embryos were removed. Opening the broad end of the eggshells, the embryos that appeared healthy were gently removed. For histochemical and immunohistochemical techniques, three embryos were used, while another three were used for TEM. Prior to fixing, the right and left caudofomralis muscles were carefully extracted. According to previous studies, muscle samples were collected during the early embryonic stages when muscle development and growth takes place^19,23^.

### Fixation

Samples used for light microscopic examination was immediately preserved in 10% neutral buffered formalin and immersed in Bouin's solution for half an hour. Subsequently, the fixed samples underwent alcohol and ethanol dehydration at increasing concentrations (70%, 80%, 90%, and 100%). Methyl benzoate was then used to clean the samples. Following that, samples that had been dehydrated were impregnated and embedded in Paraplast (MilliporeSigma, St. Louis, MO, USA). Table 1 lists the paraffin-embedding processing times for the samples.

**Table 1:** The processing time of the samples in paraffin embedding techniques.

Age process	5 d	8 d	15 d
1-Fixation			
A-NBF	8 h	13 h	24 h
B_Bouin’s solution	1/2 h	1/2 h	1/2 h
2-dehydration Alcohol70%I	2 h	2 h	2 h
Alcohol 70%II	2 d	2d	2d
Alchol70%III Alchol80%	1 h	2 h	2 h
Alchol90%	1 h	2 h	1 h
Alchol100%	1/2 h	1/2 h	1/2 h
Alchol100%	1/2 h	1/2 h	1/2 h
3-clearing with methylebenzot			
MBI	1 h	1 h	1 h
MB II	12 h	12 h	12 h
MBIII	12 h	12 h	12 h
4-embedding in paraffin			
P I	2 h	2 h	1/2 h
P II	2 h	2 h	1/2 h
PIII	4 h	4 h	1 h

NBF, neutral buffer formalin; h, hours; d, days; MB I, methyl bonzoate1, MB II, methyl benzoate II; PI, paraffin I; P II, paraffin II; P III, paraffin III.

### Histological examination

Serial 5-µm transverse and longitudinal slices were cut with a Leica RM2125 microtome (Leica Microsystems, Wetzlar, Germany). The sections were then maintained at 40 °C in an incubator to ensure they remained dry. The sections were stained for a general histological investigation using hematoxylin and eosin^24^.

### Preparations of resin embedding samples for semi-thin sections

The resin-embedding method made use of Karnovsky’s fixative. The fixative was prepared in the manner described below^25,26^: 10 mL of each of the following are combined: 30% distilled water, 50% glutaraldehyde, 25% paraformaldehyde, and 50 mL phosphate buffer. Samples from embryos on day eight were used. The neck's skin was carefully peeled off and measured between 2.0 and 3.0 mm in length. Karnovsky fixative, 4 °C overnight (Table 2).

**Table 2:** Components of the fixative.

Fixative	Components	Amount
Karnovsky Fixative	Paraformaldehyde, 25% freshly prepared	10 ml
	Glutaraldehyde 50%	10 ml
	Na-Phosphate buffer (0.1 M, pH 7.4)	50 ml
	Distilled water	30 ml
N a-Phosphate buffer (0.1 M, pH 7.4)	Solution A	
	Na2HPO4 2H2O	17.02 g
	Distilled water	600 ml
	Solution B	
	NaH2PO4 H2	6 g
	Distilled water	200 ml
	Using solution	
	Solution A	580 ml
	Solution B	219 ml
Citrate-buffer (pH 6.0)	Solution A	
	Citrate C6H8O7 H2O	21 g
	Distilled water	1 l
	Solution B	
	Sodium citrate Na3C6H5O7 2H2O	29.41 g
	Distilled water	1 l
	Using solution	
	Solution A	9 ml
	Solution B	41 ml
	Distilled water	Add 500 ml

Following osmium tetroxide postfixation, the samples were embedded in resin, washed, and crystallized in a 60 °C oven. Additionally, a pure resin/alcohol mixture was infused into them. The resin was embedded using propylene oxide (Merck, Darmstadt, Germany). A 1:1 mixture of epoxy resin and propylene oxide was then used for around 30 min, and finally, the epoxy resin mix was used for three hours. The epoxy resin composition was prepared by mixing 12 mL of dodecenylsuccinic anhydride (DSAA), 5 mL of Araldite (Huntsman Advanced Materials, The Woodlands, TX, USA), and 5 mL of EMbed 812 (Polysciences Europe GmbH, Eppelheim, Germany). After the samples were embedded, the epoxy resin mixture was heated to 60 °C to polymerize the samples. Next, an accelerator was added to the mixture (2,4,6-Tris[dimethylaminomethyl]phenol; 1.5%). The blocks were incubated at three different temperatures: 60, 70, and 75 °C^24,27,28^. Using an ultramicrotome Ultracut E (Reichert-Leica, Germany), semithin sections were cut at 1 μm and stained with methylene blue and toluidine blue^19,23^. Semithin sections were dyed after the resin was dissolved in a saturated alcoholic solution of sodium hydroxide. The stained slices were examined using a Leitz Dialux 20 microscope and a Canon PowerShot A95 digital camera.

### Immunohistochemistry staining

#### Immunohistochemistry staining procedures for CD34, CD68, and MMP-9

Following the manufacturer’s instructions, anti-polyvalent horseradish peroxidase/3,3′-diaminobenzidine (DAB), a ready-to-use reagent (Thermo Fisher Scientific, Waltham, MA, USA), was used to achieve antigen localization using the avidin–biotin complex technique in the Lab Vision Ultra Vision Detection System^29^. The procedures were carried out following^30–34^.

The 5-µm-thick paraffin sections were cleaned for 5 min in a pH 7.4 phosphate-buffered solution (PBS). Subsequently, they underwent xylene dewaxing and were rehydrated using ever-higher ethanol and alcohol grades. The slices were stored in hydrogen peroxide blocks at room temperature to inhibit endogenous peroxidase activity. After that, the parts were given one further 10 mins of running water beneath the faucet. The slides were treated for 20 min at 95–98 °C in a water bath with a 10-mmol sodium citrate buffer (pH 6.0; Table 2) to improve antigen retrieval. After leaving the slides cool for 20 min at room temperature, PBS was used three times for 5 min each time to clean them (pH 7.4). Using Thermo Fisher Scientific’s Ultra V Block, nonspecific background staining was blocked for 5 min at room temperature. This was done to avoid staining the artifact by restricting the staining time to no more than 10 min. After the sections were incubated at 4 °C for a whole night with the primary antibody (Table 3) applied, PBS (pH 7.4) was used to wash the sections three times for 5 min each.

**Table 3:** Identity, sources, and working dilution of antibodies used in Immunohistochemical studies.

	Supplier	Origin	dilution	Incubation	Antigen retrieval	Biotinylated secondary antibodySupplier
Anti- MPP9	Thermo Fischer Scientific, Lab vision Corporation, Fremont, USA	Mouse (mc, Ab-1) Clone D(33)376 Rabbit polyclonal	1:30	Over night	boiling in citrate buffer (pH 6.0), 20 min	Goat anti polyvalent
CD34	MOUSE ANTI CHICKEN CD34 (Bio rad)	MOUSE ANTI CHICKEN CD34 Monoclonal Antibody (Clone: AV138) (Cat.no MBS224490)	1:100	Over night	Boiling in citrate buffer (pH 6.0), 20 min	Goat anti-Mouse IgG (H + L) Secondary Antibody Catalog # 31569 Dilution; 1:100 1 h at room temperature
CD68 (Macrophage Marker) Ab-3 (Clone KP1)	Mouse Anti-CD68 Thermo Fisher Scientific Lab Vision Corporation, Fremont, USA	Mouse Monoclonal Antibody Cat. #MS-397-R7	1:100	Over night	Boiling in citrate buffer (pH 6.0), 20 min	
VEGF	Rabbit anti -VEGF (Invitrogen by Thermo Fisher Scientific Waltham, MA, USA))	Rabbit VEGF Polyclonal Antibody (clone: RB-222- P0) (Cat.no PA1- 21796)	1:100	Over night	Boiling in citrate buffer (pH 6.0), 20 min	Goat anti-rabbit secondary antibody (cat. no. K4003, EN Vision+ TM System Horseradish Peroxidase Labelled Polymer; Dako) Ready to use 30 min at room temperature
CD21	Rabbit Anti- CD21 antibody (Abcam)	Rabbit Anti-CD21 antibody monoclonal [SP186](ab227662)	1:100	Over night	Boiling in citrate buffer (pH 6.0), 20 min	

Antibodies used that showed reactivity in avian species.

The sections were coated with the secondary antibody (Table 3) and left to remain at room temperature for 10 min. Following three 5-min PBS washes (pH 7.4), the slices were left at room temperature for 10 min to be incubated with a streptavidin-peroxidase combination (Thermo Fisher Scientific UK and Lab Vision Corporation). Two milliliters of DAB plus substrate and one drop of DAB plus chromogen were mixed, applied to the sections, and left to remain at room temperature for 5 min to view the bound antibodies. The incubation process was carried out in a humid room. After applying the counterstain, Harris hematoxylin, it was left for 30 s. The sections were dehydrated for 5 min in 100% ethanol twice, after which they were washed in xylene and covered with a DPX (dibutylphthalate polystyrene xylene) mounting solution. We utilized a Leitz Dialux 20 microscope (Leitz GmbH, Oberkochen, Germany) and a Canon PowerShot A95 digital camera (Canon Inc., Tokyo, Japan) to analyze the immunohistochemical staining.

#### Immunohistochemical procedures for vascular endothelial growth factor (VEGF)

For the two-step immunohistochemical staining process 33, Agilent Technologies, Inc., Santa Clara, California, USA, used the Dako EN Vision + Single Reagent (HRP. Mouse). We applied the staining method that Abdo and associates developed. In summary, paraffin-embedded sections five micrometers thick were dewaxed, rehydrated, and rinsed three times with PBS (pH 7.4), each for 5 min. Methanol was treated with drops of 3% hydrogen peroxide and left to stand at room temperature for 20 min to inhibit endogenous peroxidase activity. After that, it was given a 10-min rinse under running water. To remove antigen, slides were placed in a 10-mm sodium citrate buffer (pH 6.0; Table 2) and heated in a tap water bath for 20 min to a temperature of 95–98 °C. After that, the slides were left to cool at ambient temperature for an additional 20 min. Following that, the sections were washed three times for 5 min each using PBS (pH 7.4). To avoid nonspecific background staining, drops of Dako Protein Block (Agilent Technologies, Inc.) were applied to each segment and allowed to settle at room temperature for 5 min. It should be noted that less staining than anticipated may occur after extended blocking. The sections were subsequently treated with the primary antibody (a distinct type of antibody used in a recent publication that showed reactivity in avian species)^35^.

Table 3 contains a list of all the antibodies used in immunohistochemistry research, along with their names, sources, and working dilutions. Slides were incubated with the secondary antibody for 30 min at room temperature before being washed three times for 5 min each using PBS (pH 7.4) (Table 3). After three 5-min rinses in PBS (pH 7.4), the slides were once again treated with DAB and substrate-chromogen for 5–10 mins at room temperature. This causes a brown precipitate to be produced at the antigen site. Harris hemoxoxylin was used as a counterstain for 30 s on the sections. After being washed in xylene and coated with DPX, the sections were subjected to two rounds of dehydration, lasting 5 min each in 90% and 100% ethanol. As before, we used the Leitz Dialux 20 microscope and the Canon PowerShot A95 digital camera to assess immunohistochemical staining. To create negative control samples, we used the same procedure but without the main antibody.

### Statistical analysis

The data obtained from the IHC studies of CD34, VEGF, CD68, and CD21 were analyzed statistically using Graph-Pad Prism (GraphPad 8.0.1 Software, San Diego, CA, USA). The differences that were found to be significant between CD34, VEGF, CD68, and CD21 were analyzed using one-way analysis of variance (ANOVA), and p-values less than 0.05 were considered significant. To compare the means of various receptors pairwise, Tukey’s multiple-range test was used.

The study was conducted in compliance with the Animals in Research: Reporting In Vivo Experiments (ARRIVE) guidelines. All methods were performed in accordance with the relevant guidelines and regulations^35^.

### Supplementary Information

Supplementary Figure 1.

## Data Availability

The data sets collected and/or analyzed during the current study are available from the corresponding authors on reasonable request.
