# Supplementary material for: Immunohistochemical properties of embryonic telocytes in a myogenic microenvironment
Source: Sci Rep. 2024 May 27;14:12034. doi: 10.1038/s41598-024-62103-1 (PMC11130138; doi:10.1038/s41598-024-62103-1)
Supplement: Supplementary file 1 — Supplementary Figure 1. [file 41598_2024_62103_MOESM1_ESM.pdf]

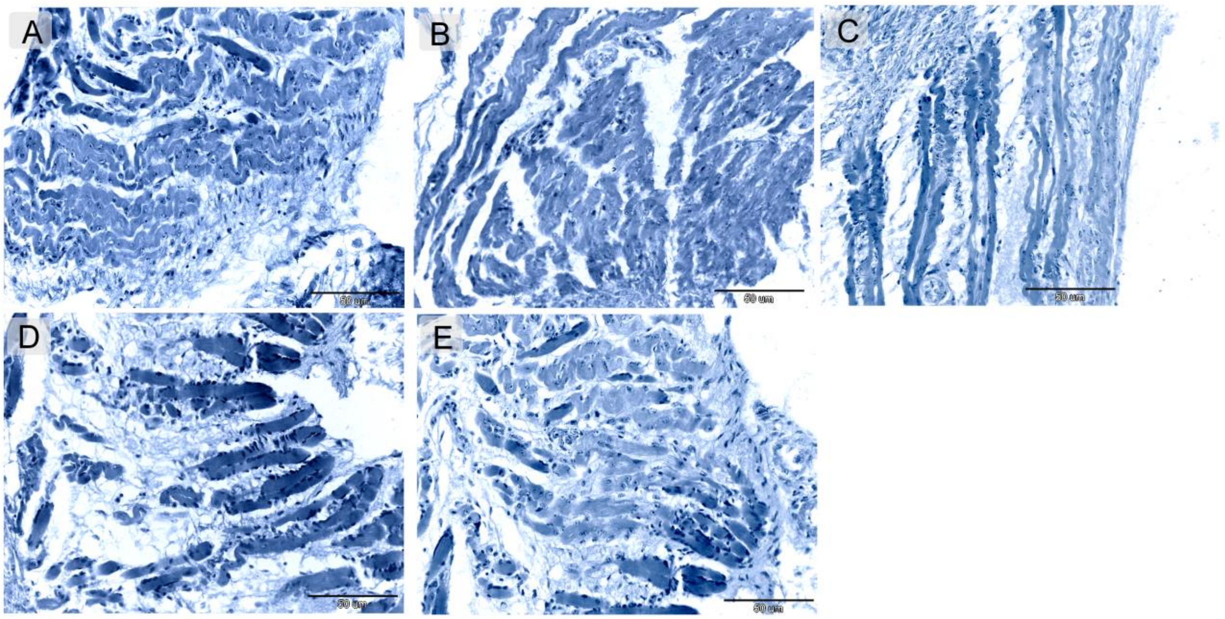

Negative control of IHC. (A) Negative control for CD34. (B) Negative control for VEGF. (C): Negative control for MMP-9. (D): negative control of the CD68. (E): negative control of the CD21.
